# Impact of different climatic conditions on peak core temperature of elite athletes during exercise in the heat: a Thermo Tokyo simulation study

**DOI:** 10.1136/bmjsem-2022-001313

**Published:** 2022-06-23

**Authors:** Lennart P J Teunissen, Kaspar M B Jansen, Emiel Janssen, Boris R M Kingma, Johannus Q de Korte, Thijs M H Eijsvogels

**Affiliations:** 1 Department of Design Engineering, Delft University of Technology, Delft, The Netherlands; 2 Department of Training & Performance Innovations, TNO, Unit Defence, Safety & Security, Soesterberg, The Netherlands; 3 Radboud Institute for Health Sciences, Department of Physiology, Radboud University Medical Center, Nijmegen, The Netherlands

**Keywords:** Heat stress, Thermoregulation, Athlete, Olympics

## Abstract

**Objectives:**

To evaluate how separate and combined climatic parameters affect peak core temperature during exercise in the heat using computer simulations fed with individual data.

**Methods:**

The impact of eight environmental conditions on rectal temperature (T_re_) was determined for exercise under heat stress using the Fiala-thermal-Physiology-and-Comfort simulation model. Variations in ambient temperature (T_a_±6°C), relative humidity (RH±15%) and solar radiation (SR+921 W/m^2^) were assessed in isolation and combination (worst-case/best-case scenarios) and compared with baseline (T_a_32°C, RH 75%, SR 0 W/m^2^). The simulation model was fed with personal, anthropometric and individual exercise characteristics.

**Results:**

54 athletes exercised for 46±10 min at baseline conditions and achieved a peak core temperature of 38.9±0.5°C. Simulations at a higher T_a_ (38°C) and SR (921 W/m^2^) resulted in a higher peak T_re_ compared with baseline (+0.6±0.3°C and +0.5±0.2°C, respectively), whereas a higher RH (90%) hardly affected peak T_re_ (+0.1±0.1°C). A lower T_a_ (26°C) and RH (60%) reduced peak T_re_ by −0.4±0.2°C and a minor −0.1±0.1°C, respectively. The worst-case simulation yielded a 1.5±0.4°C higher T_re_ than baseline and 2.0±0.7°C higher than the best-case condition.

**Conclusion:**

Combined unfavourable climatic conditions produce a greater increase in peak core temperature than the sum of its parts in elite athletes exercising in the heat.

**Key messages**

**What is already known**

- The magnitude of exercise-induced increases in core temperature and subsequent performance loss or health risks depends on the severity of climatic conditions.
- Elite athletes are recommended to participate in heat preparedness programs before competition in hot and humid environments, such as the Tokyo 2020 Olympic Games. Still, actual climatic conditions may vary from the simulated conditions.

**What are the new findings**

- Realistic variations in air temperature, relative humidity (RH) and solar radiation (SR) have distinct effects on the estimated peak core temperature of elite athletes exercising in the heat.
- The worst-case scenario simulation (T_a_ 37.5°C: RH: 79%, SR: 921 W/m^2^) predicted a peak T_re_ of 40.3±1.0°C after 46±10 min of high-intensity exercise, with 56% of the athletes demonstrating a T_re_ >40°C and 17% a T_re_ >41°C.
- The combination of unfavourable climatic conditions led to a synergistic response with a greater effect on T_re_ than the sum of its parts, highlighting the added value of computer modelling beyond physiological testing in an environmental chamber.

## Introduction

The Tokyo Olympic and Paralympic Games were organised under challenging climatic conditions. Analyses of historical data revealed that an ambient temperature (T_a_) of 31.3±3.1°C and relative humidity (RH) of 59%±10% were expected around noon,^1^ whereas solar radiation (SR) could exceed 900 W/m^2^. These environmental conditions accelerate exercise-induced increases in core temperature (T_c_),^2^ which is known to negatively impact exercise performance^3–7^ and increase the risk for the development of heat-related illnesses.^8^ Therefore, preparatory testing for Tokyo 2020 was recommended by experts,^9^ and many countries implemented heat preparation programmes for their Olympic teams.^10–12^

The Dutch Olympic Team launched the Thermo Tokyo program, in which individual heat tolerance was determined during an exercise test in simulated Tokyo conditions.^13^ Although such tests provide useful insight into the responses to a hot humid climate in general, climatic conditions may vary from day to day, and changes in T_a_, RH and SR are known to impact human thermoregulation.^14^ As it is not feasible to subject elite athletes to exercise testing across a large variety of climatic conditions, we aimed to evaluate the impact of various Tokyo summer climatic conditions on T_c_ responses using computer simulations of the individual exercise tests, fed with individual athlete data.

## Methods

### Study cohort and design

This is a substudy of the Thermo Tokyo project of which the rationale, design and measurements have been described in detail previously.^13^ In short, Dutch outdoor athletes (≥16 years) were recruited via TeamNL infrastructures. All athletes were active at the elite level as they competed at international tournaments and top-level competitions.^15^ Detailed anthropometric measurements were conducted, and all athletes completed an incremental cycling test until volitional exhaustion in simulated Tokyo conditions (T_a_ 32°C, RH 75%). Data from 54 participants (14 endurance-trained, 8 mixed-trained, 12 power-trained and 20 skill-trained athletes) were used to feed the thermophysiological simulation model in order to assess the impact of distinct climatic conditions on peak T_c_.

### Data collection

Anthropometric characteristics were derived from a DXA scan (Discovery-A, Hologic, Bedford, USA) and a full-body 3D scan (Artec 3D, Luxembourg). Height, weight, fat percentage and characteristics of 24 body segments were derived from these measurements. Gastrointestinal temperature (T_gi_) and power output (W) were continuously measured during the exercise test using the myTemp system^16^ and a bicycle ergometer (Lode B.V., Groningen, Netherlands) or Tacx Neo Smart T2800 (Tacx B.V., Wassenaar, Netherlands), respectively. Finally, the time to exhaustion of the incremental exercise test was established.

### Computer simulation

The validated Fiala-thermal-Physiology-and-Comfort (FPC) simulation model (V.5.4)^17 18^ was used to model the impact of eight different environmental conditions (table 1) on rectal temperature (T_re_). Individual characteristics (sex, age, VO_2max_, acclimatisation status), anthropometrics and exercise intensities during the test (metabolic equivalent of task score (METs), calculated from power output)^19^ served as input for the model. First, the baseline condition was modelled to reproduce the physiological responses collected during the exercise test in the climatic chamber. Then, simulations with adjustments in T_a_ (±6°C), RH (±15%) and SR (+921 W/m^2^) were conducted. Finally, a best-case and worst-case scenario simulation was conducted based on the 95% CI boundaries for the expected T_a_, RH and SR at noon during the Tokyo Olympics.^1^ Wind speed was kept constant at exercise test conditions in the climatic chamber (standard airflow of 0.2 m/s; convective cooling by pedalling movements was accounted for by an adjusted airflow of 1.0 m/s at the legs). An average gross efficiency factor of 0.2 was used. Standardised clothing settings were selected for shirt and shorts. Peak T_re_ was used as the primary outcome. The reduction in the time to reach the peak T_re_ attained in the baseline simulation was included as a secondary outcome parameter.

**Table 1:** Summary of the eight distinct environmental conditions that were entered into the Fiala-thermal-Physiology-and-Comfort simulation model

Simulations	T_a_ (°C)	RH (%)	V_air_ (m/s)	Rad (W/m^2^)
Baseline	32	75	0.2	0
Higher T_a_	38	75	0.2	0
Lower T_a_	26	75	0.2	0
Higher RH	32	90	0.2	0
Lower RH	32	60	0.2	0
Radiation	32	75	0.2	921
Worst case	37.5	79	0.2	921
Best case	25.1	39	0.2	0

Rad, radiation; RH, relative humidity; T_a_, ambient temperature; V_air_, air flow; W, watt.

## Results

Fifty-four elite athletes (53.7% female) participated in this Thermo Tokyo substudy, and all data were collected successfully. Characteristics of the study cohort are summarised in table 2.

**Table 2:** Characteristics of the complete cohort as well as sex-specific groups

	Total cohort	Male athletes	Female athletes	Endurance trained athletes	Power trained athletes	Skill trained athletes	Mixed trained athletes
N	54	25	29	14	12	20	8
Age (year)	25±4	25±4	25±4	23±5	23±3	25±4	29±4
VO_2max_ (mL/kg/min)*	41±11	43±11	39±11	56±6	40±4	31±4	40±3
Height (cm)	178±10	185±9	171±6	174±10	176±8	178±7	186±15
Weight (kg)	75±13	83±11	69±10	64±10	79±10	78±11	81±13
Fat content (%)	18±6	13±5	22±5	16±5	15±6	23±5	15±6
Time to exhaustion (min)	46±10	43±10	48±8	50±10	44±5	44±11	45±10
Peak power output (W)*	190±51	226±47	160±32	274±50	240±51	170±25	251±57

*Peak power output and subsequently derived VO_2max_ values have been attenuated due to the long trial duration, strenuous climatic conditions and athlete sport type.^13^

### Model performance

A peak T_gi_ of 38.9±0.5°C was measured at the end of the exercise test in the environmental chamber. Computer simulations using the FPC model yielded an estimated peak T_re_ of 38.8±0.6°C at baseline conditions (T_a_ 32°C, RH 75%, no SR). Accordingly, the difference between the measured versus simulated peak core temperatures was −0.1±0.5°C.

### Impact of climatic conditions

**Figure 1:**
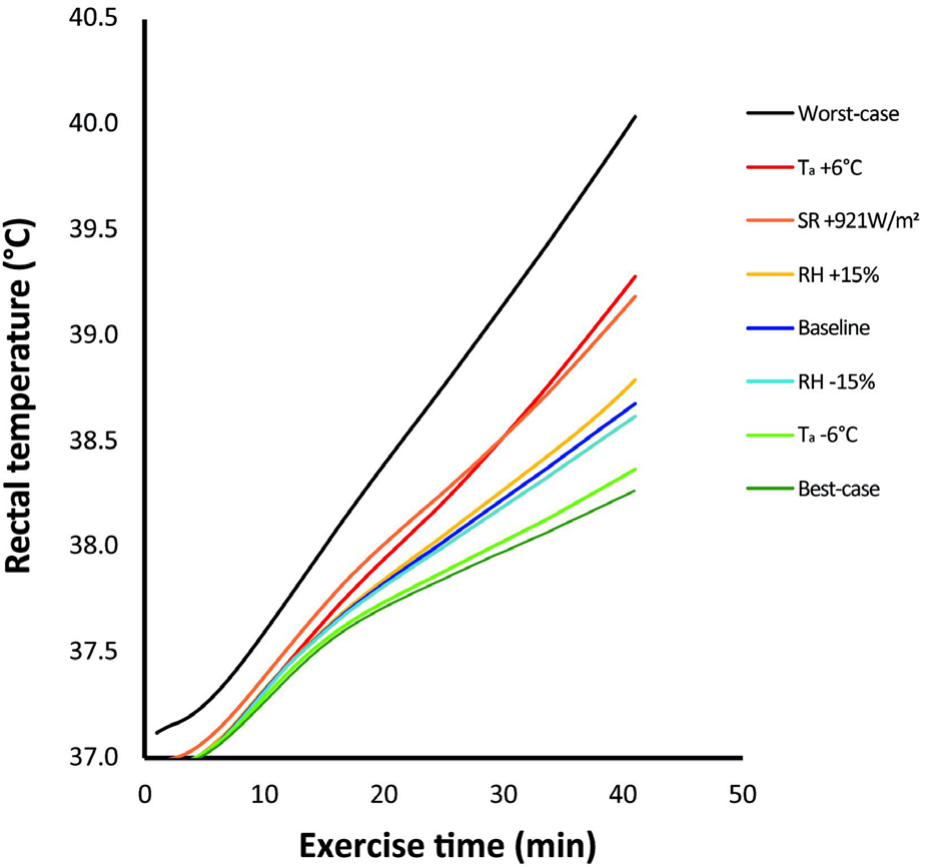
Typical outcome of the computer simulation of a single participant, with exercise-induced increases in rectal temperature for each of the eight simulated climatic conditions. T_a_, ambient temperature; RH, relative humidity; SR, solar radiation.

**Figure 2:**
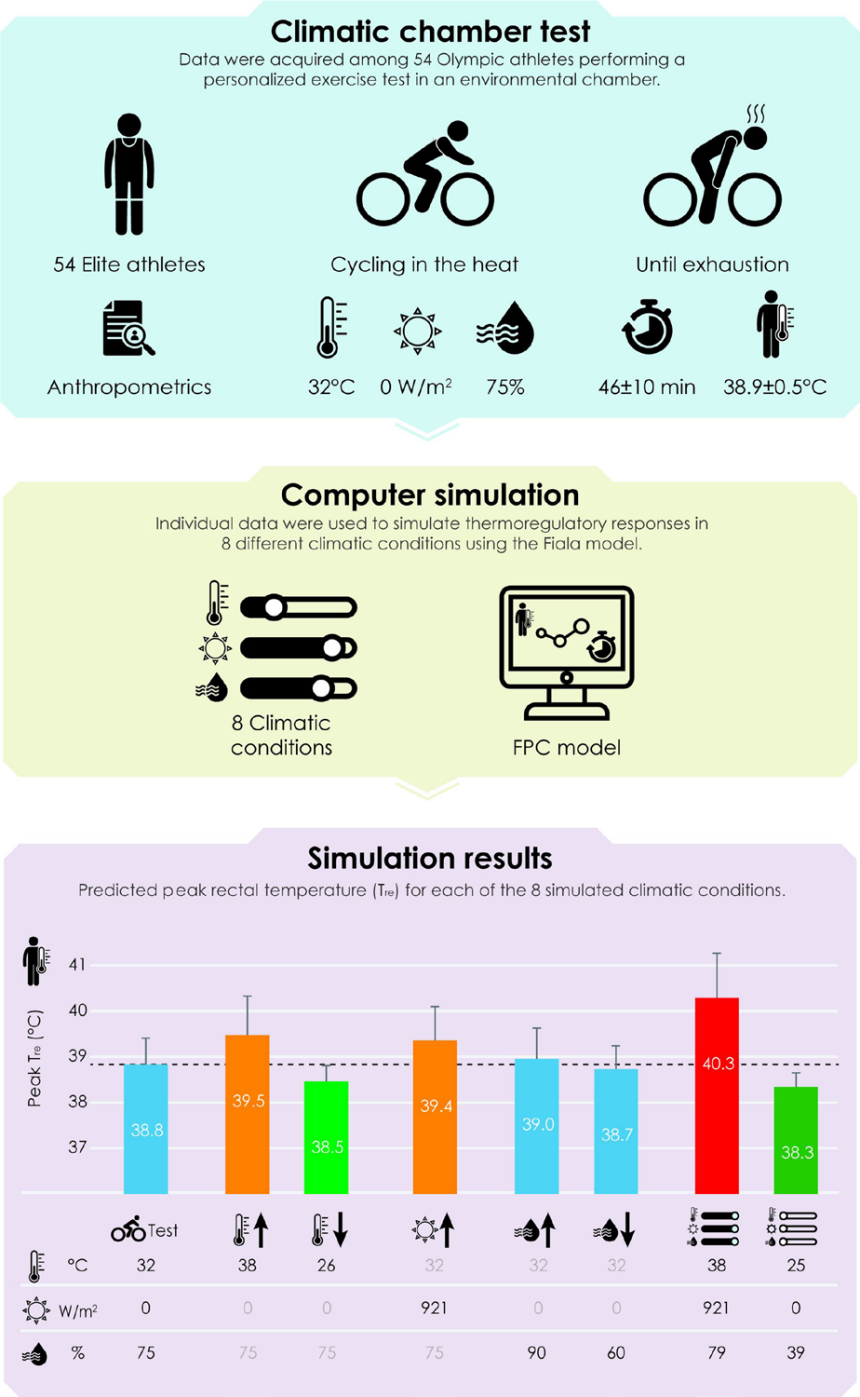
Outcomes of the computer simulation of eight distinct climatic conditions on peak rectal temperature (T_re_) of elite athletes exercising in the heat. An isolated increase in air temperature and solar radiation led to a higher peak T_re_, but the impact of the worst-case scenario on peak T_re_ was greater than the sum of its parts. FPC, Fiala-thermal-Physiology-and-Comfort.

A typical example of exercise-induced changes in T_re_ of an individual athlete for each of the eight climatic conditions in the FPC simulations is shown in figure 1. On a group level, a higher T_re_ was predicted following exercise in the condition with a 6°C higher T_a_ (+0.6±0.3°C) and the condition with maximal SR (+0.5±0.2°C) compared with the baseline condition. Peak T_re_ of the baseline simulation was attained 9±7 and 9±8 min earlier in these respective conditions. A 15% increase or decrease in RH had little effect on peak T_re_ (+0.1±0.1°C and −0.1±0.1°C, respectively), and the time to reach baseline peak T_re_ was only 2±9 min shorter in the +15% condition. A 6°C lower T_a_ resulted in a lower peak T_re_ than baseline (−0.4±0.2°C). Peak T_re_ was 1.5±0.4°C higher in the worst-case scenario compared with the baseline condition, with an absolute predicted value of 40.3±1.0°C. As many as 56% of the athletes had a peak T_re_>40°C, and 17% had a peak T_re_>41°C. Furthermore, peak T_re_ was attained 19±6 min (41%±13%) earlier during exercise in worst-case conditions. The best-case simulation yielded a 0.5±0.3°C lower T_re_ than baseline and 2.0±0.7°C lower T_re_ than the worst-case condition. An overview of all FPC model predictions is summarised in figure 2.

## Discussion

This simulation study showed that small to moderate variations in climatic conditions could considerably impact the peak T_re_ of elite athletes exercising in the heat. A 6°C increase in T_a_ or the addition of high-intensity SR contributed to an accelerated exercise-induced T_re_. In contrast, the impact of variations in RH on peak T_re_ was limited. Beyond the effect of isolated climatic conditions, the worst-case model highlighted that a combination of heat stressors leads to more significant heat stress, evidenced by predicted peak T_re_ values >40°C in 56% of the elite athletes. Therefore, the outcomes of this study provide important insight into the thermoregulatory responses of elite athletes during exercise under various challenging climatological conditions.

### FPC model

We used the FPC model to simulate the impact of different climatic conditions on T_re_. After feeding the model with physiological and anthropometric data for every individual athlete that participated in the present study,^7 13^ the average difference between the measured and predicted T_re_ for baseline climatic conditions was acceptably small (−0.1±0.5°C). Hence, we used these FPC settings to simulate the remaining conditions.

### Climatic conditions

We found a substantial variability of peak T_re_ across the simulated climatic conditions. The greatest increase in peak T_re_ was observed at a 6°C increase in T_a_. This finding aligns with a recent study in which T_a_ was the most important climatic parameter to affect peak performance during endurance running events.^20^ We also found that the impact of T_a_ on predicted T_re_ was not linear, as a 6°C decrease in T_a_ had less effect on peak T_re_ compared with a 6°C increase in T_a_ (−0.4±0.2 vs +0.6±0.3). These observations stress the added value of computer simulations beyond exercise tests with physiological measurements, as proper predictions can optimise heat mitigation strategies across multiple scenarios of climatic conditions. Maximal SR appeared to have a large effect on peak T_re_. This is in line with previous research, indicating a significant increase in the body’s heat gain with increasing radiation intensity.^21^

In contrast to our hypothesis and existing literature,^5^ we found little impact of variations in RH on peak T_re_. This may be due to the small magnitude of RH variations (±15%) and/or the low wind speed, limiting the possibility to evaporate sweat. Indeed, variations in RH between 52% and 71% have previously been shown not to result in significant changes in T_re_ during a 1-hour steady state run with little air flow.^22^ In addition, a change in skin temperature may potentially affect the impact of RH variations on heat loss. For example, increases in skin temperature at +15% RH or decreases in skin temperature with −15% RH may attenuate the heat loss effect due to the altered water vapour pressure difference between skin and environment. This could have contributed to the low impact of RH level in the current study but needs to be confirmed in future studies as skin temperature was unavailable in our simulations.

### Practical application

We showed that computer modelling of exercise-induced increases in T_re_ provides additional insights beyond physiological testing in an environmental chamber. Hence, this approach may be adopted in preparation for future World Championships, Olympic Games and other exercise events in environmentally stressful conditions. Computer simulations provide athletes and coaches unique insight into changes in individual thermophysiological and performance indicators across different climatic scenarios without the burden of extensive exercise testing. Hence, this information can be used to select the most appropriate precooling/per-cooling strategies,^23^ hydration plan^24^ and race strategy.

### Strengths and limitations

Strengths of the study include the large sample size (n=54) and a large number of simulations (8 distinct conditions) to assess the impact of variations in the Tokyo climate on the predicted T_re_ of Olympic athletes. This study, however, is also subject to limitations. First, we assumed a similar exertional heat production across climatic conditions, whereas athletes will generally adjust their pacing in an anticipatory way dependent on conditions,^25^ potentially affecting their physiological responses. Further, the wind speed was kept stable in this study, whereas it would have been interesting to explore the thermal impact of sport-specific wind speeds. Nevertheless, our results highlight the impact of different realistic climatic scenarios on the thermal strain, and the consequences if preparatory measures and anticipatory signals are not taken seriously.

## Conclusion

Isolated climatic conditions, such as a higher T_a_ and high SR, significantly increase peak T_re_ in elite athletes exercising in the heat. Moreover, the combination of unfavourable climatic conditions in a worst-case scenario led to a synergistic response with a greater effect on T_re_ than the sum of its parts.
